# Supplementary material for: MetaRibo-Seq measures translation in microbiomes
Source: Nat Commun. 2020 Jun 29;11:3268. doi: 10.1038/s41467-020-17081-z (PMC7324362; doi:10.1038/s41467-020-17081-z)
Supplement: Supplementary file 10 — Supplementary Data 7 [file 41467_2020_17081_MOESM10_ESM.zip › File2/Confidence_High_Taxonomy/408144_out.krona.html]

Javascript must be enabled to view this page.

members
magnitude
magnitudeUnassigned
count
unassigned
taxon
rank

408144\_out

2

2
superkingdom
2

1
phylum
1224

1
class
1236

1
order
91347

family
1
543

561
1
genus

1
species
562

SRS1055067\_contig\_number\_16798

1
phylum
1239

1
class
91061

186826
order
1

family
1
81852

1350
1
genus

1
species

SRS1055099\_contig\_number\_9776
1352
